# Vitamin D and Disease Severity in Multiple Sclerosis—Baseline Data From the Randomized Controlled Trial (EVIDIMS)

**DOI:** 10.3389/fneur.2020.00129

**Published:** 2020-02-25

**Authors:** Priscilla Bäcker-Koduah, Judith Bellmann-Strobl, Michael Scheel, Jens Wuerfel, Klaus-Dieter Wernecke, Jan Dörr, Alexander Ulrich Brandt, Friedemann Paul

**Affiliations:** ^1^Charité—Universitätsmedizin, Corporate Member of Freie Universität Berlin, Humboldt—Universität zu Berlin, Berlin Institute of Health, NeuroCure Cluster of Excellence, Berlin, Germany; ^2^Experimental and Clinical Research Center, Max Delbrück Center for Molecular Medicine, Berlin, Germany; ^3^Department of Neurology, Charité—Universitätsmedizin Berlin, Berlin, Germany; ^4^Department of Neuroradiology, Charité—Universitätsmedizin Berlin, Berlin, Germany; ^5^Department of Biomedical Engineering, Medical Imaging Analysis Center, Universitätsspital, Basel, Switzerland; ^6^Institute of Biometry and Clinical Epidemiology, Charité -Universitatsmedizin Berlin, corporate member of Freie Universität Berlin, Humboldt—Universität zu Berlin, Berlin Institute of Health, Berlin, Germany; ^7^CRO SOSTANA GmbH, Berlin, Germany; ^8^Multiple Sclerosis Center Hennigsdorf, Oberhavel Clinics, Berlin, Germany; ^9^Department of Neurology, University of California, Irvine, Irvine, CA, United States

**Keywords:** multiple sclerosis, vitamin D, vitamin D deficiency, T2w lesion count, EDSS score, disease severity, EVIDIMS

## Abstract

**Objective:** To investigate the associations between hypovitaminosis D and disease activity in a cohort of relapsing remitting multiple sclerosis (RRMS) and clinically isolated syndrome (CIS) patients.

**Methods:** In 51 RRMS and 2 CIS patients on stable interferon-β-1b (IFN-β-1b) treatment recruited to the EVIDIMS study (Efficacy of Vitamin D Supplementation in Multiple Sclerosis (NCT01440062) baseline serum vitamin D levels were evaluated. Patients were dichotomized based on the definition of vitamin D deficiency which is reflected by a < 30 vs. ≥ 30 ng/ml level of 25-hydroxyvitamin D (25(OH)D). Possible associations between vitamin D deficiency and both clinical and MRI features of the disease were analyzed.

**Results:** Median (25, 75% quartiles, Q) 25(OH)D level was 18 ng/ml (12, 24). Forty eight out of 53 (91%) patients had 25(OH)D levels < 30 ng/ml (*p* < 0.001). Patients with 25(OH)D ≥ 30 ng/ml had lower median (25, 75% Q) T2-weighted lesion counts [25 (24, 33)] compared to patients with 25(OH)D < 30 ng/ml [60 (36, 84), *p* = 0.03; adjusted for age, gender and disease duration: *p* < 0.001]. Expanded disability status scale (EDSS) score was negatively associated with serum 25(OH)D levels in a multiple linear regression, including age, sex, and disease duration (adjusted: *p* < 0.001).

**Interpretation:** Most patients recruited in the EVIDIMS study were vitamin D deficient. Higher 25(OH)D levels were associated with reduced T2 weighted lesion count and lower EDSS scores.

## Introduction

The exact cause of multiple sclerosis (MS), a chronic inflammatory and neurodegenerative autoimmune disease of the central nervous system (1) is unknown. However, several environmental and genetic factors have been associated with MS pathogenesis, among them are vitamin D (VD) serum levels (2–5), vitamin D receptor polymorphisms (VDP) (6–10), and sunlight exposure (11, 12). In fact, increasing evidence suggests that VD deficiency may affect disease progression and outcome in MS.

VD is considered a potent immunomodulator which may reduce MS risk based on epidemiological and experimental evidence (13–15). High serum VD levels have been associated with lower MS risk, reduced relapse rates (16) and better disease outcome (2, 13, 17). Circulating VD levels are lower during relapses compared to phases of disease stability (18). *In vitro* studies also showed a reduced proliferation of CD4^+^ T cells and myelin basic protein (MBP)-specific T cells in the presence of 1,25(OH)D3, the active metabolite of vitamin D (18). The immunomodulatory effect of vitamin D in upregulating anti-inflammatory cytokines points to an important role in the homeostasis of T cells (18) hence its relevance in autoimmune diseases.

The high prevalence of hypovitaminosis D worldwide (19, 20) and the continuously increasing incidence of MS incidence (21) highlights the need to investigate possible associations.

The American Institute of Medicine (IOM) (22), the Japan Endocrine Society (23), the Osteoporosis Council of Canada (24), and the International Osteoporosis Foundation (25) have suggested divergent cut-offs for vitamin D deficiency ranging from < 20 to < 30 ng/ml. These definitions are mainly based on the prevention of osteoporosis (22, 23, 26–28). In healthy populations, serum vitamin D levels ≥ 30 ng/ml are considered sufficient (29–31). In the past decade, several clinical interventional trials have investigated the effect of vitamin D supplementation on the pathogenesis of MS, among them the EVIDIMS (NCT01440062) (32), the SOLAR (33), the VIDAMS (34), and the CHOLINE trials (35). Moreover, data from observational studies suggest that there may be a beneficial interaction of vitamin D and interferon-β regarding their immunomodulatory effect (36, 37).

In this paper, we dichotomized patients enrolled in the EVIDIMS study at baseline prior to randomization and vitamin D supplementation according to their serum 25(OH)D levels and based on VD deficiency. We then investigated possible associations of the VD status with clinical and MRI parameters. The EVIDIMS study investigated the effect of oral VD supplementation on clinical and MRI parameters in MS patients on stable immunomodulatory interferon-β treatment. The primary outcomes of the EVIDIMS trial are presented elsewhere (38). The goal of this analysis is to investigate if the EVIDIMS cohort is representative of a VD deficient RRMS cohort by (a) investigating VD serum levels and (b) investigating correlations with disease severity.

## Methods

### Patients

The cohort in this study represents the baseline cohort of phase II interventional EVIDIMS trial (NCT01440062) (32) and comprised 51 patients with RRMS and 2 CIS patients, all Caucasians, according to the 2005 McDonald criteria (39). The detailed in- and exclusion criteria for the EVIDIMS trials have been published earlier (32, 38). Briefly, inclusion criteria were age of 18-65 years, Expanded Disability Status Score (EDSS) below 6.5, stable interferon-β-1b treatment for a minimum of 3 months, freedom of relapses for at least 30 days prior to study entry, and a relapsing remitting disease course or CIS. The exclusion criteria were other immunomodulatory therapies than interferon-β-1b, VD intake within 6 months before study entry, pregnancy or lactation and kidney disease, bone marrow dysfunction or hypercalcemia. To account for sunlight intensity as a possible confounder, patients were recruited from a single geographic region in the north-eastern part of Germany.

Disability was evaluated using EDSS scores. All clinical and MRI assessments were performed blinded to serum VD status.

### Measurement of Serum 25(OH)D Levels

Serum 25(OH)D levels were measured by the Bioscientia Institute for Medical Diagnostics GmbH (Berlin, Germany) using the LIAISON® chemiluminescence analyzer, DiaSorin (Dietzenbach, Germany).

### Definitions of Groups

Subgroup analyses based on serum 25(OH)D levels were performed based on the suggested cut-offs for VD levels for bone health as proposed by the American Institute of Medicine, the Japan Endocrine Society and the global definition for vitamin D deficiency and sufficiency. Hence, dichotomous analyses comparing serum 25(OH)D levels of (< 30 vs. ≥ 30 ng/ml) were performed. These groups were compared with respect to the following (i) MRI imaging parameters: T2 weighted lesion count (T2C), T2 lesion volume (T2V), white matter volume (WM), gray matter volume (GM), total brain volume and (ii) clinical parameters such as EDSS scores.

### MRI Protocol

The same MRI machine and protocol were used in all patients using a Magnetom TIM TRIO 3 Tesla MRI (Siemens, Healthineers, Erlangen, Germany). High resolution images were acquired using a sagittal three-dimensional (3D) T2-weighted (T2w) SPACE sequence (repetition time (TR) ms /echo time (TE) ms/ inversion time (TI) ms (5,000/502/900; flip angle 9°/, isotropic resolution 1 mm3, Generalized Autocalibrating Partially Parallel Acquisition (GRAPPA 2), a 3D SPACE-FLAIR (TR ms/TE ms/ TI ms, 6,000/388/2,100) with similar spatial parameters and a three-dimension (3D) T1w, magnetization-prepared rapid gradient-echo (MP-RAGE) sequence (TR ms/echo time ms/ TI ms, 1,900/3.03/900; flip angle 9°, isotropic resolution 1 mm3, GRAPPA 2). Five minutes after injection of 0.1 mmol/kg gadolinium-labeled diethylenetriaminepentaacetic acid (Gd-DTPA, Magnevist, Bayer-Schering, Berlin, Germany), a 3D T1w gradient recall echo volumetric interpolated breath-hold examination (VIBE) sequence (TR ms/TE ms, 4.8/2.2; flip angle 9°, isotropic resolution 1 mm3, GRAPPA 2) was applied. The quality of acquired images was reviewed, and raw data were transferred to a Linux workstation and processed semi-automatically using an image coregistration (FMRIB's Linear Image Registration Tool, FMRIB Analysis Group, University of Oxford, Oxford, UK) and inhomogeneity correction routine embedded into the MedX v.3.4.3 software package (Sensor Systems Inc., Sterling, VA, USA).

The MedX v.3.4.3 software package was used for measuring the white matter lesion load and lesion count of T2w scans, the number and volume of contrast-enhancing and hypointense lesions on T1w scans. Segmentation of brain lesions was performed semi-automatically using the lesion segmentation toolbox (LST) (40) lesion probability algorithm on FLAIR images with subsequent manual correction using ITK-SNAP (41). Normalized brain volume (NBV) and percentage brain volume change (PBVC) were obtained with SIENA (FMRIB library) (40).

### Statistical Analyses

Results for continuous variables are expressed as median with 25 and 75% quartiles [25, 75%] for non-normally distributed data and as mean ± standard deviation (SD) for age and disease duration. Results for categorical variables are given as absolute numbers and relative frequencies (%). Due to the non-normally distributed continuous data and small sample sizes, exact Mann–Whitney *U*-test was used for the comparison of independent groups. Simple linear regression analyses were used for the association between EDSS and serum vitamin D levels. Multivariate linear regressions were applied in order to adjust this association for possible confounding factors such as age, disease duration, and sex. We also tested for collinearity between the independent variables. Non-parametric analysis of covariance was applied to test T2-weighted lesion counts and volume for differences between groups of vitamin D sufficiency (< 30 vs. ≥ 30 ng/ml), adjusted for the covariates age, gender, and disease duration. A *p*-value of < 0.05 was considered significant. All other tests should be understood as constituting exploratory data analysis, such that no adjustments for multiple testing have been made. Statistical analyses were performed in R version 3.4.2 (2017-09-28), IBM© SPSS© Statistics, Version 24, © Copyright 1989, 2016 SPSS Inc., an IBM Company and SAS version 9.4 [TS1M3] Copyright © 2002 by SAS Institute Inc., Cary, NC, USA.

## Results

### Patients and Serum 25(OH)D Levels

Data from all 51 RRMS and 2 CIS patients recruited into the EVIDIMS trial were used for this study. The mean age of patients was 43 ± 10.2 years with 37 (69.8 %) females and a mean disease duration of 9 ± 9.3 years.

The median (25, 75% Q) serum 25(OH)D was 18 ng/ml (12, 24), signifying overall VD deficiency (Table 1). After dichotomization by serum 25(OH)D < 30 vs. ≥ 30 ng/ml, 48 out of 53 patients (91%) had 25(OH)D < 30 ng/ml with a median (25, 75% Q) of 15 ng/ml (10, 21) in the < 30 ng/ml group while the remaining 5 out of 53 patients (9%) had 25(OH)D ≥ 30 ng/ml with a median of 33 (33, 35) ng/ml (*p* < 0.001) (Table 2).

**Table 1:** Baseline characteristics of 51 RRMS and 2 CIS patients recruited in the EVIDIMS Trial.

**Variable**	**Total**
Female *n* [%]	37 (69.8)
Age in years [mean ± SD]	43 ± 10.2
Disease duration in years [mean ± SD]	9 ± 9.3
Serum 25 (OH) [med (25, 75% quartiles)] ng/ml	18 (12, 24)
EDSS [med (25,75% quartiles)]	2.0 (1.5, 3.0)
T2 lesion count [med (25,75% quartiles)]	55 (33, 84)
T2 lesion vol (ml) [med (25,75% quartiles)]	4 (2, 9)

**Table 2:** Characteristics of patients with 25(OH)D levels < 30 vs. ≥ 30 ng/ml.

**Subgroup analysis of 25(OH)D levels** **<** **30 vs**. **≥** **30 ng/ml**			
**Variable**	**< 30 ng/ml** **(*n* = 48)**	**≥ 30 ng/ml** **(*n* = 5)**	***p*****-value**^**1**^
Age in years [mean ± SD]	42.9 ± 10.1	39 ± 12.8	0.46
Disease duration in years [mean ± SD]	9.6 ± 6.7	5.7 ± 5.4	0.15
Serum 25(OH) [med (25, 75% quartiles)] ng/ml	15 (10, 21)	33 (33, 35)	<0.001
EDSS [med (25, 75% quartiles)]	2.5 (1.5, 3)	1.0 (1, 2)	0.04
T2 lesion count [med (25, 75% quartiles)]	60 (36, 84)	25 (24,33)	0.03
T2 lesion vol (ml) [med (25, 75% quartiles)]	4 (2, 10)	2 (2, 3)	0.13
Gray matter vol (ml) [med (25, 75% quartiles)]	597 (553, 620)	594 (557, 652)	0.63
White matter volume(ml) [med (25, 75% quartiles)]	536 (509, 591)	567 (547, 570)	0.49
Brain volume(ml) [med (25, 75% quartiles)]	1126 (1063, 1209)	1161 (1114, 1222)	0.53

1 *Mann–Whitney test*.

### Association of Serum 25(OH)D and Disability Scores

**Figure 1:**
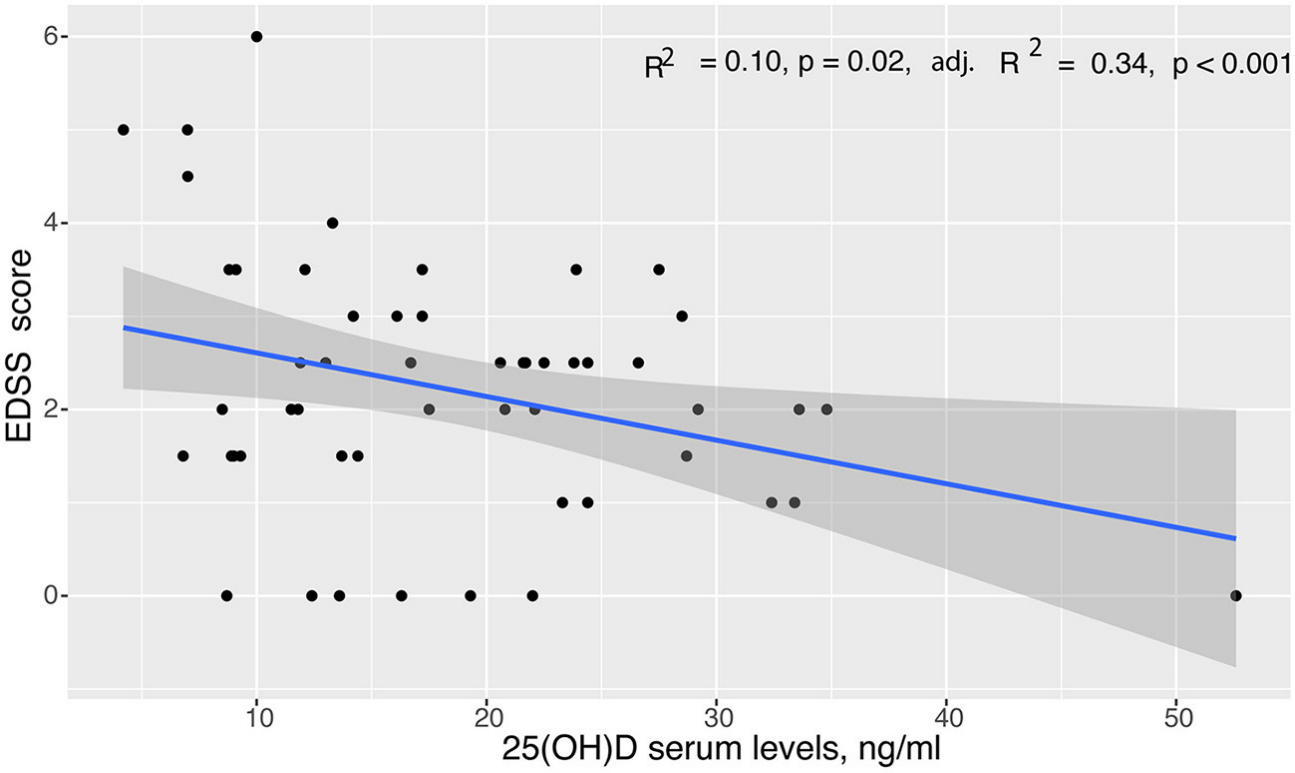
Linear regression analysis of EDSS scores to serum vitamin D levels in the entire cohort with 95% confidence limits. Simple Linear regression showed a significant association between EDSS scores and serum 25(OH)D levels (*p* = 0.02), which was confirmed after adjustment for age, sex, and disease duration in multiple linear regression analysis (adjusted *p* < 0.001).

In the entire cohort, there was an inverse association between EDSS score and serum 25(OH)D level. The results remained the same after adjustment for age, sex, and disease duration in a multivariate analysis (simple: *R*^2^ = 0.10, *p* = 0.02; adjusted: *R*^2^ = 0.34, *p* < 0.001) (Figure 1). EDSS was higher in patients with deficient (< 30 ng/ml) 25(OH)D: 2.5 (1.5, 3) compared to 1.0 (1, 2) for vitamin levels ≥ 30 ng/ml (*p* = 0.04) (Table 2). There was no collinearity between the predictor variables tested.

### Association of Serum 25(OH)D and MRI Parameters

The median (25, 75% Q) T2 weighted lesion count was 25 (24, 33) in the ≥ 30 ng/ml group, compared to 60 (36, 84) in the < 30 ng/ml group (*p* = 0.03; adjusted for age, gender, 25(OH)D levels, and disease duration: *p* < 0.001). The numerical difference in median T2 lesion volume [2 (2, 3) ml in the ≥ 30 ng/ml group; 4 (2, 10) ml in the < 30 ng/ml group] was not significant [*p* = 0.13; adjusted for age, gender, 25(OH)D levels, and disease duration: *p* = 0.06] (Table 2). We found no association of serum vitamin D levels with gray matter, white matter, and total brain volumes (Table 2).

## Discussion

Using baseline characteristics of patients recruited in the EVIDIMS trial, we show that according to the definitions of the American Society of Medicine and the Japan Endocrine Society (22, 23, 27), VD deficiency is common in RRMS/CIS patients living in the north-eastern part of Germany. Moreover, the range of serum levels and the proportions of serum VD levels is in line with previously published MS cohorts. In this cohort, VD deficiency showed an inverse association with measures of disability and was furthermore linked to a higher T2w lesion count but was not associated with T2 lesion volume, total brain volume as well as gray and white matter volumes.

A high prevalence of VD deficiency in MS patients has been demonstrated in other studies: In an earlier study on a different patient cohort, we reported VD deficiency already in very early phases of MS (5). A cross-sectional study of 50 RRMS patients revealed a VD deficiency with a mean of 22.3 ng/ml /ml (42). In a Moroccan study of 113 MS patients, 97.3% of patients were VD deficient with a mean of 11.69 ng/ml (43). There is an ongoing debate whether VD deficiency represents a risk factor for MS or whether this association is rather due to reverse causality, i.e., low VD levels are a result of MS, for example as a consequence of reduced outdoor activities. The fact that the cohort investigated here was relatively young with a rather mild disability status may be an argument against reverse causality. Moreover, in an earlier study on a different cohort of early MS or CIS patients we also observed VD deficiency already in very early phases of MS (5) which also supports the interpretation that VD deficiency is rather a cause than a consequence of MS.

Also, the inverse association between disability measures and serum 25(OH)D levels demonstrated here are in line with previous reports: a population-based study on 136 MS patients from Australia showed that patients with higher disability (EDSS > 3) had a higher probability of insufficient vitamin D levels (44). Similarly, a cross-sectional study on 267 MS patients revealed an inverse association between EDSS scores and serum 25(OH)D levels in the entire cohort (45).

The association of deficient serum vitamin D levels with MRI activity is controversial. While some studies could not show any associations between serum 25(OH)D levels and MRI activity (46) others showed that the development of T2w or contrast enhancing lesions is correlated to the VD serum level (47). Our MRI data support the association of MRI activity and VD status. We, however, did not detect any association between VD levels and measures of both global and regional brain atrophy. Although speculative, this might well be due to the small sample size and the low power to detect such associations.

Taken together, our results confirm the high prevalence of VD deficiency in MS patients and the possible associations it has with MRI and clinical disease activity. Of note, causality or directionality of these associations cannot be inferred from ours and other cross-sectional studies reporting similar associations. Specifically, it is possible that reduced VD leads to a more severe disease course. Alternatively, it may also be possible that higher disability may lead to lower VD levels, e.g., by less physical activity and reduced sunlight exposure. Previously, we reported reduced VD levels in a cohort of very early MS/CIS patients with low disability, which makes the latter less likely, but not impossible. Our study, however, has some important limitations: first, the overall small sample size and particularly the very small number of patients in the VD sufficient group result in a low power to detect associations. Secondly, our analyses are based solely on the cut-offs for deficient and sufficient serum VD levels defined for osteoporosis and normal health which may not be generalized to the MS population. Additionally, we only investigated patients from a particular area of Germany which increased the homogeneity of our sample on the one side but on the other side might not be representative for MS patients in general. Finally, as serum samples were taken throughout the year, seasonal variations of VD levels may confound data.

In conclusion, the EVIDIMS cohort is representative of a typical RRMS cohort with VD deficiency, we confirm previous associations of low serum VD with clinical and disease activity which provides further support for the role of VD in the development and progression of MS.

## Data Availability Statement

The datasets generated for this study are available on request to the corresponding author.

## Ethics Statement

The study was reviewed and approved by the German Federal Institute for Drugs and Medical Device (BfArM, 4037578) and the local ethics committees (11/0386-ZS EK 13). All patients gave written informed consent before entering the study.

## Author Contributions

PB-K collected, processed, cleaned, and prepared the data for statistical analysis, performed the statistical analysis, interpreted the data, and drafted the manuscript. JB-S recruited patients and collected clinical data. MS acquired and processed MRI data. JW acquired and processed MRI data. K-DW was a responsible biometrician, performed the statistical analyses, and interpreted the data. JD designed the trial, drafted the study protocol, recruited patients and generated data, and drafted the manuscript. AB and FP designed and conceptualized the study, interpreted the data, and revised the manuscript for intellectual content.

### Conflict of Interest

PB-K is funded by the DFG Excellence grant to FP (DFG exc. 257) and is an Einstein Junior scholar. JB-S received travel grants and speaking fees from Bayer Healthcare, Biogen, Merck Serono, Sanofi-Aventis/Genzyme, Teva Pharmaceuticals, and Novartis. MS reports no conflict of interest. JW is CEO of MIAC AG Basel, Switzerland. He served on scientific advisory boards of Actelion, Biogen, Genzyme-Sanofi, Novartis, and Roche. He is supported by grants of the EU (Horizon2020), German Federal Ministries of Education and Research (BMBF) and of Economic Affairs and Energy (BMWI). JD received research support by Bayer and Novartis, travel support by Bayer, Novartis, Biogen, Merck Serono, and honoraria for lectures and advisory by Bayer, Novartis, Biogen, Merck Serono, Roche, Sanofi Genzyme. AB is cofounder and shareholder of Motognosis and Nocturne. He is named as an inventor on several patent applications regarding MS serum biomarkers, OCT image analysis and perceptive visual computing. FP reports research grants and speaker honoraria from Bayer, Teva, Genzyme, Merck, Novartis, MedImmune and is a member of the steering committee of the OCTIMS study (Novartis), all unrelated to this work. K-DW is the owner of the company SOSTANA GmbH, Berlin, Germany.
